# Psychometric Evaluation of Patients with Psoriasis and their Association with Patients’ Psychological Characteristics

**DOI:** 10.31138/mjr.030125.epr

**Published:** 2026-01-08

**Authors:** Konstantinos Kontoangelos, Charitomeni Vavouli, Ioanna Daikidou, Sofia Tsiori, Sofia Martinaki, Stavros Karageorgiou, Charalabos Papageorgiou, Alexander Stratigos

**Affiliations:** 1 1st Department of Psychiatry, Eginition Hospital, Medical School National & Kapodistrian University of Athens, Athens, Greece;; 2 University Mental Health Neurosciences and Precision Medicine Research Institute “Costas Stefanis”, Athens, Greece;; 3 1st Dermatology Department, Andreas Syggros Hospital for Skin Diseases, National & Kapodistrian University of Athens, Athens, Greece;; 4 Department of social work, University of West Attica, Athens, Greece;; 5 Medical School National & Kapodistrian University of Athens, Athens, Greece

**Keywords:** psoriasis, psychosomatic, personality, depression, hostility

## Abstract

**Objectives:**

The onset, progression and recurrence of psoriasis is believed to be related to mood and psychological disorders, such as depression. Psoriasis affects the personal, social and sexual life of patients resulting in psychological stress.

**Aim:**

The purpose of the research is the psychometric evaluation of patients with psoriasis.

**Methods:**

Seventy one patients with psoriasis were enrolled in the study. The measurement of the severity of psoriasis in the patients was implemented through specific indicators, the Psoriasis Area and Severity Index (PASI). Beck’s Depression Inventory (BDI) was used to measure the intensity of depressive symptoms. In addition, the Eysenck Personality Questionnaire (EPQ), the self-completed scale Brief Symptom Inventory SCL - 90, and the HDHQ questionnaire (Psychometric Hostility and Direction of Hostility Questionnaire) were given. The validity and reliability of questionnaires rely on input from experts and potential responders who may suggest pertinent revisions to prepare forms with attractive designs, easily understandable questions, and correctly ordered points that appeal to target respondents.

**Results:**

Females with psoriasis have on average significantly higher scores in the BDI depression scale (13.5±10.0 vs. 7.9±8.3, p=0.009) compared to men, as well as in the SCL90 depression scale (13.64±10.18 vs. 7.00±5.45, p=0.003). The extroversion scale of the EPQ is statistically significantly related with the patients’ psychiatric history and stable income. Patients receiving medication for psoriasis are 2 times more likely to answer the lie questions positively than patients not receiving medication (OR=2.01, p=0.028).

**Conclusion:**

Psoriasis exerts a direct influence on the social daily life of the individual, at a functional and behavioural level. It is essential to emphasise the importance of addressing the psychological effects of psoriasis along with its physical aspects for better outcomes.

## INTRODUCTION

Skin diseases such as psoriasis can significantly affect the patient’s self-image, self-esteem and quality of life.^1^ Psoriasis is a multifactorial inflammatory condition with a disease burden that extends beyond the physical symptoms experienced by patients and affects all aspects of quality of life, including physical, psychological, social, sexual, and occupational parameters. Data suggest that the social stigma, high levels of stress, physical limitations, depression, work problems, and other psychosocial comorbidities experienced by patients with psoriasis are not always proportional to or predicted by other measures of severity of disease, such as plaque severity.^2,3,4,5^ Higher levels of psoriasis-related stressors and reductions in quality of life are significantly associated with suicidal ideation in patients with psoriasis,^6,7,8,9,10^ hostility,^11^ low self-esteem, anxiety, or depression and have problems dealing with negative emotions. Pruritus is a very common symptom of psoriasis, affecting more than 70% of people and for many patients it is the most bothersome symptom of the disease.^12,13^ Living with psoriasis can cause negative emotions, which can affect quality of life.^14^ The relationship between stress and psoriasis has been described as a vicious cycle. Psoriasis itself can act as a stressor for patients. Psoriasis is a disease with a strong social stigma. ^15^ Regarding personality, psoriasis patients show more intense neuroticism^16^ they are less open to new experiences compared to the rest of the research participants^17^ while those who had psoriasis earlier in life had stronger evidence of hostility, irritability and reduced trust towards others.^18^ The purpose of this study is to evaluate psychometric characteristics in patients with psoriasis. and the correlation between them in order to delve further into the psychopathology of patients with psoriasis.

## MATERIALS AND ΜETHODS

### Participants

The population study consisted of patients 71 patients with psoriasis of ages between 24 and 85 years old, who are being monitored at the psoriasis outpatient clinic at the Venereal and Skin Diseases Hospital of Athens “Andreas Syggros” who had or were currently suffering from psoriasis. The sample was taken based on the administration of questionnaires. The research in question is a cross-sectional study, which lasted for two months. No participants were removed from the sample. In order to collect the data, anonymous questionnaires were filled out in paper form, in person. The participants were informed about the purposes of the study and their participation was voluntary, while anonymity and confidentiality were maintained. All patients were selected randomly .The entire process is entirely governed by the principles of the Code of Ethics and Conduct. All methods were carried out in accordance with the Declaration of Helsinki (Protocol code: 257 and date of approval: 20-06-2023). Patients provided their informed written consent to participate in this study and for the publication of the results. Official permissions obtained for re-use of questionnaires.

### Means of Data Collection

#### Brief Demographic Information Questionnaire and clinical characteristics of the participants

Information was requested regarding age, sex, weight, smoking, employment, educational level, marital status, number of children, place of origin, place of residence, co-morbidity, date of disease onset, the date of diagnosis, the medical specialties visited, the part of the body where there is a skin lesion, the treatment they have received for psoriasis, the history of hospitalisation for the disease, the existence of psychiatric history, diagnosis, medication use, and whether they are undergoing psychotherapy.

#### The Symptom Checklist-90 (SCL-90)^19^

The questionnaire is self-completed and measures 9 psychopathology parameters (as many as its subscales), which are: (1) somatisation; (2) depression; (3) anxiety; (4) phobic anxiety; (5) obsessive compulsive; (6) paranoid ideation; (7) psychoticism; (8) hostility; and (9) interpersonal sensitivity. The questionnaire includes 90 questions in total.^20^

The Beck Depression Inventory (BDI) is a 21-question multiple-choice self-assessment report inventory, one of the most widely used instruments for measuring the severity of depression. Its development marked a shift among health care professionals, who had until then viewed depression from a psychodynamic perspective, instead of it being rooted in the patient’s own thoughts.^21^

#### Psychometric Personality scale of extraversion, neuroticism, psychoticism (Eysenck Personality Questionnaire, EPQ) (Eysenck 1975) ^22^

The Eysenck personality questionnaire consists of 84 entries evaluated by the patient with a yes or no answer. The purpose of this questionnaire is to explore four dimensions of personality: psychoticism (P), neuroticism (N) extraversion (E), and lying (L).^23^

#### Psychometric Hostility and Direction of hostility Questionaire (HDHQ) ^24^

The psychometric scale of hostility and direction of hostility consists of 51 entries, is self-completed. It consists of four subscales: (a) intropunitiveness, (b) extrapunitiviveness, (c) direction of hostility, and (d) general hostility. The assessment instrument consists of five subscales: (a) the act out hostility, (b) criticism of others, (c) delusional or paranoid hostility, (d) self-criticism, and (e) delusional guilt. The first three subscales are summed to form an extrapunitive score and the other two are summed to yield an intropunitive score.

#### PASI SCORE (Psoriasis Area and Severity Index)^25^

The PASI score is a measurement tool that dermatologists commonly use to assess the extent and severity of psoriasis. Using the tool also allows them to monitor the progression of the condition and evaluate the effectiveness of treatment. The scoring involves rating the symptoms of psoriasis from none to very severe and estimating the percentage of the body that they affect. Researchers also use PASI scores to determine the effectiveness of psoriasis medications in clinical trials. The range of absolute PASI scores is 0–72, with higher scores indicating a greater severity of psoriasis.

### Statistical analysis

The categorical variables are presented with frequencies and percentages and the numerical variables are described by mean value, standard deviation, minimum and maximum value. Kolmogorov-Smirnov test was applied to investigate if the scales of depression (BDI), psychopathology (SCL90), personality (EPQ), and hostility (HDHQ) follow a normal distribution. The null hypothesis of normality was rejected for all scales, therefore nonparametric tests were applied to examine the relationships of psychometric scales with demographic and clinical patient characteristics. Specifically, the Mann-Whitney test was applied to examine the relationship of the psychometric scales with binary categorical variables. The Spearman correlation coefficient was used to examine the relationship of the psychometric scales with the age of the patients and the age of onset of the disease.

A forward variable selection method was applied to all models, incorporating all patients’ demographic and clinical characteristics. Only variables that were found to be statistically significant are included in the final models, apart from age and gender, which were retained in all models regardless of significance

The BDI scale was used as a categorical variable and logistic regression was applied to select patient characteristics associated with patients experiencing mild or moderate depression versus minimal depression.

The SCL90 psychopathology scales were combined into a psychopathology index to examine the patients’ demographic and clinical characteristics that influence the psychopathology index. Each scale of the SCL90 was transformed into a binary variable taking the value 0 when the score was less than the median and 1 when it was greater than or equal to the median. The psychopathology index is the sum of the 9 dichotomous variables and takes values from 0 (when a patient scores below the median on all SCL90 scales) to 9 (when a patient scores above the median on all SCL90 scales). The higher values a patient scores, the more symptoms of psychopathology they exhibit. We assume that the psychopathology index follows the binomial distribution with 9 independent tests (as a “success” is defined when a patient scores higher than the median on any of the 9 questions), and therefore, binomial logistic regression was applied to examine patient characteristics that influence the psychopathology index.

The EPQ and HDHQ scales are derived from the sum of dichotomous questions, so higher scores correspond to higher levels of psychoticism, neuroticism, extraversion, and lie for the personality scale and also higher levels of hostility for the hostility scale Binomial logistic regression was used to examine the demographic and clinical characteristics of the patients affecting the EPQ and HDHQ scales. Statistical analysis was performed with the IBM SPSS Statistics 24 program and the level of significance was set at 5%. Additionally, the R statistical package was used to address the issue of overdispersion by performing quasi-binomial regression models.

## RESULTS

The mean age of patients was approximately 58.3 years with a standard deviation 14.2. Slightly more than the half of the patients were men (53.5%), 62.0% of the patients were married and the rest were single, divorced or widowed (38.0%) and more than half of the patients (59.2%) had no children. The majority of the patients had a fixed income (73.2%, **Table 1**).

**Table 1.** Demographic and clinical characteristic of the patients.

			**Frequency**	**%**
**Gender**	Female		33	46.5
	Male		38	53.5
**Family status**	Single, divorced or widowed		27	38.0
	Married		44	62.0
**Children**	Yes		29	40.8
**Income**	Not constant		19	26.8
	Constant		52	73.2

**Comorbidities**	Yes		47	66,2
**Psychiatric history**	Yes		21	29,6
**Medication for psoriasis**	Yes		64	90,1
**Skin lesions**	Head		52	73,2
	Extremities		68	95,8
	Body		56	78,9
	Genitals		31	43,7
**Index PASI**	<5		51	71,8
	>=5		20	28,2

	M	SD	Min	Max

**Age**	58.3	14.2	24	85
**Age at the onset of psoriasis**	41.9	18.7	1	80

M: mean value, SD: standard deviation, Min: minimum, Max: maximum.

Regarding the clinical characteristics of the patients, 66.2% of the patients have comorbidities, 29.6% have a psychiatric history, 90.1% of patients are taking medication for psoriasis. Patients have skin lesions on different parts of their body, with the extremities being the most commonly affected part (95.8%), followed by the trunk (78.9%), the head (73.2%), and the genitals (43.7%). The majority of the patients (71.8%) have a PASI score below 5, indicating a mild to moderate severity of psoriasis., whereas 28.2% of patients have greater severity of psoriasis. The age of the patients at the onset of the disease ranges from 1 to 80 years, with a mean value of 41.9 years and a standard deviation of 18.7 (**Table 1**).

Comparing the psoriatic patients’ psychometric scales with their gender, it turns out that females have on average statistically significantly higher levels of depression compared to males. More specifically, females with psoriasis have on average significantly higher scores in the BDI depression scale (13.5±10.0 vs. 7.9±8.3, p=0.009), as well as in the SCL90 depression scale (13.64±10.18 vs. 7.00±5.45, p=0.003). Besides that, female patients score on average significantly higher on the scale of obsessive compulsiveness (10.0±6.8 vs. 6.3±4.6, p=0.012) and delusional guilt (1.9±1.5 vs. 1.3±1.5, p=0.014) compared to male patients with psoriasis.

Patients without children have on average statistically significantly higher scores in the scales of interpersonal sensitivity (5.2±5.4 vs. 2.3±3.6, p=0.003), phobic anxiety (2.1±3.8 vs. 0.4±1.3, p=0.001), and paranoid ideation (3.0±4.0 vs. 1.6±3.0, p=0.011) compared to patients with children. Patients with psoriasis without comorbidities score significantly higher in the scale of extraversion of the EPQ scale compared to patients with comorbidities (12.8±5.9 vs. 9.6±6.4, p=0.028). On the other hand, psoriatic patients with comorbidities score statistically significantly higher on the scale of lie compared to patients without comorbidities (14.1±3.0 vs. 12.2±3.9, p=0.024). Besides that, patients with comorbidities have on average statistically significantly higher scores on the delusional guilt scale compared to psoriatic patients without concomitant diseases (1.7±1.6 vs. 1.1±1.4, p=0.036).

The patients’ psychiatric history affects the scores of depression, phobic anxiety, neuroticism, extraversion and delusional guilt. In particular, patients with a psychiatric history score statistically significantly higher on the BDI scale of depression (16.3±9.7 vs. 8.1±8.4, p=0.001) and significantly lower on phobic anxiety (0.6±2.2 vs. 1.7±3.5, p=0.028) than those without a psychiatric history. Patients with a psychiatric history have on average higher scores on the neuroticism subscale of EPQ (13.1±4.3 vs. 9.7±4.3, p=0.002) and significantly lower scores on the extraversion (7.5±7.1 vs 12.1±5.6, p=0.011) than those without a psychiatric history. Besides that, the psychiatric history is related with higher scores on the delusional guilt scale (2.1±1.6 vs 1.3±1.4, p=0.027).

It turns out that the severity of psoriasis affects the urge to act out hostility scale. In particular, patients with higher PASI scores on the PASI index (>5) score statistically significantly lower in the hostility scale than the patients with lower levels of severity of psoriasis (4.0±1.1 vs. 4.7±1.3, p=0.016).

The correlation of the psychometric scales with the patients’ age and the age of onset of the disease is presented in **Table 2**. The age of the patients is statistically significantly correlated with a positive correlation coefficient with the BDI depression scale (r=0.417, p<0.001), the SCL90 scales of somatisation (r=0.298, p=0.012), and depression (r=0.286, p=0.016). Moreover, the patients’ age is correlated positively coefficients with the EPQ scale of lying (r=0.274, p=0.021) and the scale of self-criticism (r=0.312, p=0.008) and negatively with extraversion (r=−0.289, p=0.014). Older patients tend to score higher on the scales of depression, somatisation, lying, self-criticism and lower on the scale of extraversion. The age of onset of the disease is statistically significantly related to the scale of psychoticism (either in the SCL90 scale r=0.244, p=0.040, or in the EPQ scale r=0.314, p=0.008).

**Table 2.** Correlation coefficients of the psychometric scales with age and age at the onset of disease.

		**Age**		**Age at the onset of psoriasis**	

		**Cor. Coef.**	**p**	**Cor. Coef.**	**p**
**ΒDI**		0.417	<0.001	0.217	0.069
**SCL90**	Somatization	0.298	0.012	0.198	0.097
	Obsessive-Compulsive	0.165	0.170	0.073	0.543
	Interpersonal sensitivity	−0.007	0.956	0	0.997
	Depression	0.286	0.016	0.136	0.259
	Anxiety	0.200	0.094	0.211	0.077
	Hostility	0.175	0.145	0.126	0.295
	Phobic anxiety	0.032	0.790	0.049	0.686
	Paranoid ideation	0.016	0.895	0.078	0.519
	Psychoticism	0.221	0.064	0.244	0.040

**EPQ**	Psychoticism	0.166	0.165	0.314	0.008
	Νeuroticism	0.137	0.254	0.064	0.594
	Εxtraversion	−0.289	0.014	−0.079	0.514
	Lie	0.274	0.021	0.180	0.133

**HDHQ**	Urge to act out hostility	−0.199	0.096	−0.155	0.196
	Criticism of others	−0.045	0.711	−0.002	0.985
	Paranoid hostility	0.058	0.630	0.117	0.332
	Self-criticism	0.312	0.008	0.172	0.153
	Delusional guilt	0.174	0.147	0.035	0.769

Cor. Coef.: Spearman correlation coefficient.

**Table 3** and **Table 4** present the logistic and binomial logistic regression models, incorporating the demographic and clinical characteristics that were significantly associated with the patients’ psychometric scale scores.

**Table 3.** Logistic regression of the BDI depression scale.

	**OR**	**p**
**(constant)**	0.1	0.006
**Gender: Male**	0.1	0.005
**Age of disease onset**	1.1	0.004
**PASI ≥ 5**	5.5	0.022
**Psychiatric history : Yes**	7.7	0.008

OR: odds ratio.

Reference level: gender: female, PASI <5, psychiatric history: no.

**Table 4.** Binomial logistic regression of the patients’ psychometric scales.

	**Psychological index**		**Extraversion scale of EPQ**		**Lie scale of EPQ**		**Urge to act out hostility**		**Paranoid hostility**	
	**OR (95% C.I.)**	**p**	**OR (95% C.I.)**	**p**	**OR (95% C.I.)**	**p**	**OR (95% C.I.)**	**p**	**OR (95% C.I.)**	**p**
(constant)	0.6 (0.3–1.5)	0.309	7.4 (2.3–26.3)	0.002	1.0 (0.4–2.1)	0.890	0.6 (0.5–0.8)	<0.001	0.6 (0.2–1.6)	0.279
**Gender: Male**	0.7 (0.3–1.3)	0.205	1.1 (0.6–2.2)	0.780	0.8 (0.5–1.2)	0.266	1.1 (0.9–1.3)	0.573	0.8 (0.4–1.3)	0.354
**Age of disease onset**	1.02 (1.0–1.0)	0.027	1.0 (1.0–1.0)	0.146	1.0 (1.0–1.02)	0.051	1.0 (1.0–1.0)	0.259	1.0 (1.0–1.0)	0.543
**PASI ≥ 5**							0.8 (0.6–1.0)	0.033		
**Psychiatric history: Yes**			0.3 (0.1–0.6)	0.002						
**Children: yes**	0.3 (0.2–0.6)	0.002								
**Income: constant**			0.3 (0.1–0.7)	0.007						
**Medications for psoriasis: yes**					2.0 (1.1–3.7)	0.028			0.3 (0.2–0.7)	0.003

OR: odds ratio.

Reference level: gender: female, PASI <5, psychiatric history: no, children: no, income: not constant, medication for psoriasis: no.

Gender, age of disease onset, PASI score, and psychiatric history were found to be significant predictors of the BDI depression scale. In particular, male patients have statistically significant lower odds by 90% of experiencing mild, moderate, or severe depression than female patients with psoriasis, given that all other variables are the same (OR=0.1, p=0.005). Patients diagnosed with psoriasis at an older age are more likely to experience depression. Specifically, if the age of diagnosis with psoriasis increases by one year, the odds of the patient having mild, moderate, or severe psoriasis increases by 10%, given that all other variables remain the same (OR=1.1, p=0.004). Patients with a PASI score of 5 or more are 5.5 times more likely to experience depression (mild, moderate, or severe) than patients with a score of less than 5, when all the other variables are the same (OR =5.5, p=0.022). Also, psoriatic patients who have a psychiatric history are 7.7 times more likely to develop depression than patients without a psychiatric history (OR=7.7, p=0.008).

According to the binomial logistic regression model for the psychopathology index, age at psoriasis onset and parental status (i.e., having children or not) were found to be statistically significant factors. If age of onset increases by one year, the odds of a patient scoring above the median on any of the psychopathology scales is expected to increase by 2.0%, when all the other variables remain the same (OR=1.02, p=0.027). Patients with psoriasis who have children are 70.0% less likely to score above the median on any of the psychopathology scales than patients who do not have children (OR=0.3, p=0.002). Therefore, patients with psoriasis by older age of disease onset or without children are expected to have more psychopathology symptoms.

The extroversion scale of the EPQ is statistically significantly related with the patients’ psychiatric history and stable income. Specifically, patients with a psychiatric history are 70% less likely to respond positively to the extraversion questions than patients without a psychiatric history (OR=0.3, p=0.002). Therefore, patients with a psychiatric history are less likely to show extro-version symptoms than patients with psoriasis with no a psychiatric history. Patients with psoriasis who have a stable income are 70% less likely to respond positively to the extroversion questions than patients who do not have a stable income. Therefore, stable income appears to reduce extroversion symptoms in patients relative to patients without stable income. Medication for psoriasis was found to be statistically significant predictor of the lie scale. Patients receiving medication for psoriasis are 2 times more likely to answer the lie questions positively than patients not receiving medication (OR=2.0, p=0.028). Therefore, patients with psoriasis who receive medication would be expected to present more lying symptoms. Although no differences were reported between the groups in terms of psychiatric diagnoses, the patients with greater severity (PASI < 10) showed higher rates of discouragement (61.5%) and Type A behaviour (53.8%) than those with mild severity (17.5% and 21.1%, respectively).

Patients with a PASI ≥5 are 20.0% less likely to answer positively in the questions related to hostility intention than patients with a PASI <5 (OR=0.8, p=0.033). Therefore, patients with a higher PASI status have a reduced intention to engage in hostility relative to patients with a lower PASI status. Medication for psoriasis is found to be significantly related to paranoid hostility (OR=0.3, p=0.003).

## DISCUSSION

Psoriasis is associated with psychological factors such as depression, anxiety, as well as social problems and stress. In our research the age of onset of psoriasis is 24 - 85 years (38 male and 33 female with a mean of 41.9 years and a standard deviation of 18.69 years). According to the literature, which claims that there is a clear difference in the frequency of the disease based on gender,^26^ the statistical analysis showed higher rates of psoriasis occurring in women (46.5%) compared to men (53.5%). Most patients with psoriasis that receive therapeutic intervention, pharmacotherapeutic or phototherapy, is determined on the severity of psoriasis, as can be seen from the literature.^27^

Comorbidities and psychiatric history are common in patients with psoriasis, as there is a relationship between behaviour, and emotion and the biological activity.^28^ Something that is certified by the specific study, as patients with accompanying diseases present higher percentages on the lie scale and show higher delusional guilt. In contrast, patients with psoriasis without comorbidities show higher rates of extroversion. Patients with a psychiatric history show statistically higher rates of depression, neuroticism and delusional guilt, compared to those without a psychiatric history. Regarding the location of occurrence of psoriasis, 73.2% have psoriasis on the head, 95.8% on the extremities, 78.9% on the chest and 43.7% on the genitals. Depression can impair the quality of life, social relationships and functioning of people with psoriasis. More specifically, women with psoriasis show higher rates of depression compared to male patients with psoriasis, something that is also confirmed by the literature, citing the female gender as one of the main factors for the occurrence of depression.^30^ Men with psoriasis are statistically significantly 86% less likely than women to experience mild, moderate, or severe depression. Furthermore, women with psoriasis have increased rates of obsessive-compulsive and delusional guilt compared to men.^31^

Age was found to be statistically significantly positive for depression and for SCL90 depression subscale and it was shown that the older the psoriasis patients are, the higher rates of depression they show. More specifically, patients with psoriasis, who got sick at an older age, have a 6% higher chance of experiencing symptoms of depression. Also, the degree of depression was negatively associated with psoriasis and the quality of life domain.^32^ Patients with psoriasis had significantly more depressive symptoms and population-based studies showed that they were at least one and a half times more likely to experience depression and used more antidepressants than the control group.^33^

More than 10% of patients suffer from clinical depression and twice as many have depressive symptoms.^34^ This may be attributed to the fact that in young people and women, self-esteem and self-image are structured to a greater extent based on external appearance, given that relevant social norms prevail.

As these individuals therefore feel that they fail to conform to relevant norms due to psoriasis, they more easily develop depression.^8^ Depression can enclose an effect on personality, such as neuroticism and extroversion, and proves that the connection of psychological and pathological mechanisms can affect personality. In particular, this is related with borderline personality disorder, obsessive-compulsive and narcissistic personality disorder.^35^ Regardless of the existence of psychopathological manifestations at the personality level, these patients also show more intense neuroticism traits.^18^ There is also a relationship between hostility and psychometric scales, but this relationship differs based on several factors, such as gender, psychiatric history, and depression. More specifically, the stigma and initial aggression against patients with psoriasis leads to hostility in response to the initial reactions of others towards their external appearance.^13^ Hostility as an element of personality is accompanied by similar behaviours and practices on the part of patients with psoriasis.^36^ From the statistical analysis, it was found that patients with psoriasis have an increased risk of psychiatric comorbidities and suicidal ideation. Higher levels of psoriasis-related stressors and reductions in quality of life were significantly associated with suicidality in patients with psoriasis.^37^

The main factors that contributed to depression were: beliefs about its occurrence and importance in self-esteem, greater psychological distress and lower levels of emotional social support. Therefore, improving the belief of the patients with psoriasis about the body image by reducing its importance in their personal lives may play a role in preventing depression, especially in women.^38^ Depression is also correlated with psoriatic arthritis (PsA) who is a chronic inflammatory disease that profoundly impacts both physical and psychosocial well-being. The disease can lead to a range of emotional difficulties, including anxiety, depression, and diminished self-esteem.^39^ Likewise levels of anxiety and distress are strongly associated with disease activity and functional inability in elderly patients with rheumatoid arthritis.^40^ Patients with psoriasis are vulnerable to psychological problems, such as anxiety, due to the nature of the disease which affects on the individual’s appearance and social prejudices.^41^ There is a need to emphasise the importance of addressing the psychological effects of psoriasis along with its physical aspects for better outcomes,^42^ and patients with psoriasis may experience accelerated development of multimorbidity.^43^ Future research should focus on developing strategies to address the mental health needs of this patient population for primary prevention and early detection treatment strategies, connecting the mind to the body. Awareness of the association between psychological and pathological factors can lead to tailored treatment approaches, which include not only drug therapy but also counselling, psychotherapy and patient education. The link between personality, depression, and psoriasis points to the need for individualised treatment approaches. Treatments must take into account the psychological characteristics of individuals and include psychotherapy, psycho-education, and personality management. Research hypotheses can advance further research into how personality, depression, and psoriasis interact. Further studies can examine the exact processes that occur and offer specific directions for the treatment and management of psoriasis. The supplementary material refers to two tables.

## LIMITATIONS

This was a monocentric study conducted on 71 patients and several limitations of our study should be acknowledged. The small sample and the lack of control group and of data regarding psychiatric manifestations before psoriasis onset prevented us from exploring the potential bidirectional relationship between psychiatric comorbidities and the onset of psoriasis. Future studies should consider including these patient populations and collecting baseline psychological data to provide a more comprehensive analysis.

## Data Availability

All data can be found on 1^st^ Department of Dermatology-Venereology, Andreas Syggros Hospital, Medical School, National and Kapodistrian University of Athens.
